# The effects of a brief CBT intervention, delivered by frontline mental health staff, to promote recovery in people with psychosis and comorbid anxiety or depression (the GOALS study): study protocol for a randomized controlled trial

**DOI:** 10.1186/1745-6215-15-255

**Published:** 2014-06-27

**Authors:** Helen Waller, Tom Craig, Sabine Landau, Miriam Fornells-Ambrojo, Nedah Hassanali, Catherine Iredale, Suzanne Jolley, Paul McCrone, Philippa Garety

**Affiliations:** 1 Department of Psychology, Institute of Psychiatry, King’s College London, London, UK; 2 Department of Health Service and Population Research, Institute of Psychiatry, King’s College London, London, UK; 3 Department of Biostatistics, Institute of Psychiatry, King’s College London, London, UK; 4 South London and Maudsley NHS Foundation Trust, London, UK; 5 University College London, London, UK

**Keywords:** Anxiety, Behavioural activation, CBT, Depression, Graded exposure, Psychosis

## Abstract

**Background:**

NICE guidance states that cognitive behavioural therapy (CBT) should be offered to all patients with psychosis. However, there is a need to improve access to therapeutic interventions. We aim to train frontline mental health staff to deliver brief, structured CBT-based therapies. We have developed and piloted a manualized intervention to support people with psychosis and anxious avoidance or depression to work towards a personal recovery goal.

**Methods/Design:**

The ‘GOALS Study’ is a pilot randomized controlled trial comparing usual care plus an 8-week intervention with usual care alone. The key objective is to assess clinical feasibility (recruitment and randomization; compliance with the treatment manual; acceptability and satisfaction; progress towards goals). A secondary objective is a preliminary evaluation of efficacy. Sixty-six participants with a diagnosis of psychosis, plus symptoms of depression or anxiety will be recruited from adult mental health services. Those currently refusing medication, in receipt of CBT, or with a primary diagnosis of an organic mental health problem or substance dependency will be excluded. Following informed consent, randomization will be independent of the trial team, at a 50:50 ratio, at the level of the individual and stratified by main problem focus. Following randomization, participants allocated to the intervention group will begin the 8-week intervention with a local, trained member of staff, supervised by the study coordinator. Outcomes will be assessed blind to treatment condition at 0, 12 and 18 weeks post-randomization. The primary outcome measure for the efficacy analysis will be activity levels at 12 weeks. Secondary outcome measures include mood, psychotic symptoms, quality of life and clinical distress. A health economic analysis comparing service use in each condition will also be performed. Recruitment began in March, 2013 and is ongoing until December, 2014.

**Discussion:**

This is the first trial of the GOALS intervention. The approach is brief and staff can be readily trained in its delivery: there is therefore potential to develop a cost-effective intervention that could be widely disseminated. If the trial proves clinically feasible and demonstrates preliminary evidence of efficacy, a large multi-site trial will be warranted.

**Trial registration:**

Current Controlled Trials ISRCTN: 73188383. http://public.ukcrn.org.uk/search/StudyDetail.aspx?StudyID=13538

## Background

It is estimated that over half a million people in the UK alone suffer from psychosis. For many, recovery is impeded by high levels of distress, often resulting from persisting psychotic symptoms, stigma and social exclusion [1,2]. There is evidence for an increased prevalence of anxiety and depression in people with psychosis, in comparison with the general population [3,4]. These difficulties can prevent people from engaging in meaningful activity and from achieving valued goals. Helping people to overcome these obstacles and achieve their goals should promote recovery.

There is evidence that cognitive behavioural therapy (CBT), in conjunction with antipsychotic medication, is effective in reducing distressing symptoms and hospitalizations, in comparison with medication alone [5,6]. Consequently, the latest National Institute for Health and Care Excellence (NICE) guidance [7] states that CBT should be offered to all people with psychosis or schizophrenia. New cognitive behavioural approaches for psychosis have been developed to focus specifically on recovery-oriented outcomes, such as increased activity levels and hopefulness about the future [8]. However, given the large number of patients with psychosis and the limited availability of CBT-trained professionals, there is a need to consider ways of improving access.

Brief, evidence-based CBT approaches are available, as adopted in the UK ‘Improving Access to Psychological Therapies’ programme; namely, graded exposure and behavioural activation. These interventions are effective in reducing anxiety and depression [9-11], and are often applied in CBT for psychosis, to help with concurrent difficulties with mood, and to help manage persisting psychotic symptoms [12]. Importantly, the approaches can be readily disseminated: ‘nonspecialist’ staff can be trained to deliver behavioural activation effectively, following brief training [13,14]. However, little evidence is available regarding their efficacy in this group. Based on these evidence-based methods, we have developed a new intervention designed to help people meet valued, personal goals and improve recovery, which can be delivered by frontline mental health staff as part of the team’s package of care.

### Preliminary study

We have completed a small pre-pilot study to evaluate the feasibility of the model of delivery, and the effectiveness of the intervention [15]. Six staff members, including nurses and occupational therapists, completed training over four half-days, with ongoing group supervision from a clinical psychologist. They delivered the intervention to 12 service users, all of whom were outpatients attending early-intervention or community mental health services. All participants showed increased activity and clinical improvement, and were able to meet their goals. Both staff and service users gave positive feedback on the intervention. Participant and staff feedback was sought following completion of the study [16], which informed a number of changes to the treatment manual and training package.

### Pilot randomized controlled trial

The aim of the study is to run a pilot randomized controlled trial of this intervention, specifically designed for people with psychosis: the GOALS study (‘Getting On top of Anxiety and Low mood’). Following the same structure as the pre-pilot study, therapy will be delivered over eight weekly sessions by frontline mental health staff, after receiving brief training (2 days) and fortnightly case supervision from a clinical psychologist, to provide ongoing support and to ensure fidelity to the treatment protocol. Staff will come from a range of professional backgrounds, including nursing, occupational therapy and psychology (assistants) and are likely to have little or no previous experience in delivering CBT-based therapy. The manualized intervention aims to improve recovery, social inclusion and social functioning and reduce distress, and will be evaluated in comparison to a treatment-as-usual (TAU) control group, which will be standard community mental health care. This will inform the further development of the intervention and training and will provide information for a larger multicentre trial.

The main objective of the study is to assess the clinical feasibility of delivering the therapy package to patients with a diagnosis of psychosis and concurrent anxiety or depression. We will assess this on the basis of successful recruitment and randomization, therapist and participant compliance with the treatment manual and good levels of acceptability and satisfaction with the treatment and progress towards participants’ chosen goals. A secondary objective is a preliminary evaluation of efficacy. For this, the primary outcome is activity levels at post-intervention; our assessment time point of interest. In addition we will examine a range of secondary outcomes at both post-intervention (12 weeks) and follow-up (18 weeks). These include assessments of mood, psychotic symptoms, well-being and clinical distress. A health economic analysis, assessing service use, will also be performed, to assess the cost effectiveness of the intervention.

## Methods

### Participants and study setting

The study aims to recruit 66 participants from community psychosis teams (both early intervention and recovery) within one UK National Health Service (NHS) trust for mental health: the South London and Maudsley NHS Foundation Trust, which provides treatment for 10,000 people with psychosis. Recruitment will initially be targeted at six teams (with caseloads in excess of 150 service users per team or a potential pool of 900 service users). It is estimated that at least 40% (*n* = 360) of these service users will meet our criteria and that, at a very conservative estimate, at least 120 of these would consent to participate in a research trial, thus providing ample numbers.

Potential participants with a diagnosis of psychosis and who are identified by their clinical team as having problems in daily functioning owing to anxiety-related avoidance or depression will be invited to learn more about the study. The inclusion criteria are: diagnosis of a schizophrenia spectrum disorder or currently experiencing psychotic symptoms (for example, with diagnoses of personality disorder, bipolar disorder or psychotic depression); 18 to 65 years old (or accessing adult services); clinical levels of anxiety-related avoidance or depression on outcome measures; and a desire to increase the current level of activities. Exclusion criteria are patients not meeting the above criteria, or who are currently refusing all medication; or who are currently or recently (previous 3 months) in receipt of CBT; or who have a primary diagnosis of an organic mental health problem; or who have a primary substance dependency.

### Ethical approval

The study has been reviewed and given a favourable opinion by the London Chelsea National Research Ethics Committee (reference: 12/LO/1523).

### Interventions

#### *Treatment-as-usual (TAU) control condition*

Participants in the TAU group will continue to receive all the treatment and support they received before the start of the trial, including input from their general practitioner and psychiatrist, and will be seen by their care coordinator at least monthly. As we expect there to be some variation in delivery and take-up of TAU, all service contacts will be monitored for the trial duration. Participants in the TAU arm will be offered the intervention after the end of the trial (after four months). The post-TAU treatment does not form part of this study and no data will be collected on the participants in the TAU arm after the end of the study.

#### *Treatment as usual (TAU) + GOALS intervention*

Participants allocated to the TAU + GOALS intervention will continue to receive all the treatment and support they received before the start of the trial, including input from their general practitioner and psychiatrist, and will be seen by their care coordinator at least monthly. Following randomization, participants will receive eight weekly CBT sessions with a trained member of staff, each for around one hour (these may be slightly shorter or longer, depending on client preference and where longer sessions are needed to practice particular skills in the community), within a 12-week period. In addition, participants will receive a ‘booster’ session at one month after completion of the final therapy session (this should be at approximately 14 weeks). As described earlier, the intervention is based on two established, brief CBT interventions: graded exposure for anxious avoidance and behavioural activation for depression.

The therapy is targeted at supporting people to achieve a personally desired, behavioural recovery goal (increasing activity and social engagement), through targeting symptoms of depression or anxiety. The therapist’s manual covers both anxiety (graded exposure) and depression (behavioural activation) techniques, and these techniques are structured very similarly. The techniques chosen will depend on whether the client’s difficulties in completing a particular recovery goal arise from avoidance of anxiety (for example, particular fears or physical symptoms of anxiety) or depression-related difficulties (for example, lack of motivation or energy, interference of negative thoughts or content of voices). The manual comprises detailed session-by-session plans and client hand-outs, for example educational materials on depression and anxiety and an explanation of vicious and virtuous cycles of reduced activity levels and avoidance; guidance on setting specific, measurable attainable, relevant and time-bound (SMART) goals with clients; advice on breaking down longer-term goals into more manageable steps; setting up weekly in-session and homework tasks; and a section on troubleshooting any difficulties encountered in implementing the therapy. The sessions aim to teach service users the skills to achieve their chosen goal and to use those skills independently in the future to reach new goals. The final weekly session includes meeting with a trusted friend, carer or mental health professional (if agreed with service users) to discuss the purpose of the sessions, to reinforce achievements, and to think together about ways in which that person might be able to continue to support the client, where appropriate. Service users are then invited to set a small new goal to complete together with their support person, which is reviewed at the booster session one month later.

### Outcomes and timeline

**Figure 1:**
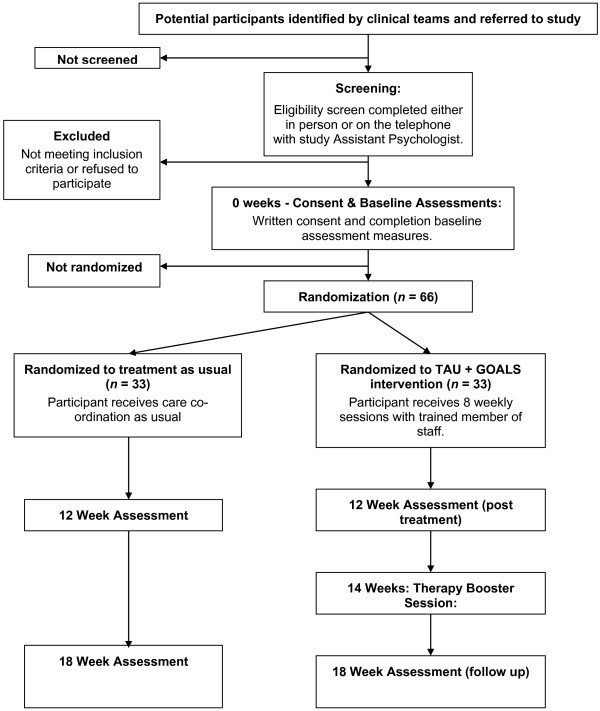
Study flowchart.

The timeline is outlined in Figure 1. Following informed consent from each participant, all outcomes will be assessed by a study researcher. Assessment time points are before randomization (‘baseline’ or 0 weeks), 12 weeks after randomization (‘post-treatment’) and 18 weeks after randomization (‘follow-up’). All outcomes will be assessed in all participants at all time points, with the exception of the Client Service Receipt Inventory (see next), which will be assessed at 0 and 18 weeks only. Baseline assessments will be completed within a time window of 4 weeks. Post-randomization assessments will have to be completed within a 4 week window, up to one week before or three weeks after the planned assessment time point. All participants (TAU and TAU + GOALS) will have a brief semistructured interview with a study assistant psychologist following completion of their final 18 week assessments. Individual goal attainment and service user consultant conducted interviews will only be undertaken in the TAU + GOALS intervention group. Fifteen participants will undertake this additional semistructured interview with a service user consultant. We will aim to purposely sample participants with a range of responses to the treatment, including those who have achieved their goals and those who have not been able to do so, following the intervention.

#### *Screening*

Screening will involve assessment using the Hospital Anxiety and Depression Scale [17], which includes clinical ‘cut-off’ scores. To be eligible to enter the study, service users must score above the clinical cut-offs (>8) on either the depression or anxiety subscales.

#### *Primary outcome*

The primary outcome measure for the study is activity level, which is assessed using the Time Budget Measure [18,19]. This is an individualized measure of activity over an average week, which was specifically designed for use with people with psychosis, and has been used in at least one trial with this population [20]. It is sensitive to change and has a high inter-rater and test-retest reliability [19].

#### *Secondary outcomes*

Psychotic symptoms will be measured using two validated and commonly used assessment measures: the Positive and Negative Syndromes Scale [21], which is a semistructured interview, looking at the presence of symptoms over the previous two weeks, and the Psychotic Symptom Rating Scales [22], which measure the dimensions of delusional and hallucinatory experience over the previous week. Anxiety and depression will be measured using the Hospital Anxiety and Depression Scale, as described earlier, plus an additional measure of anxious avoidance (the Mobility Inventory) [23], since this will be targeted in the intervention in order to support participants to work towards particular personal goals that anxiety might currently prohibit. Well-being, quality of life and general distress will all be assessed using validated assessment measures with good psychometric properties (the Warwick-Edinburgh Mental Well-being Scale [24], the Manchester Short Assessment of Quality of Life [25] and Clinical Outcomes in Routine Evaluation - 10 (CORE-10) [26]). Cost effectiveness of the intervention is an important target, if it is to be delivered in the UK NHS. The Client Service Receipt Inventory [27] is a measure developed to calculate cost effectiveness. It consists of eight items that record a client’s contact with mental health professionals and related services, such as day services, criminal justice services, or prescriptions and benefits received, over the previous four-month period. Qualitative feedback will also be collected through semistructured interviews, aiming to examine participants’ experience of the interventions and other care, blocks and enablers of delivery of the intervention, and contextual factors that might be associated with variation on outcomes.

For all participants in the intervention group, progress towards individualized client goals will be assessed weekly by the staff therapist completing the intervention, using ten-point visual analogue scales [28]. The scales were developed and piloted as part of the previous study and were found to be simple and easy to complete. Additionally, to assess the quality of treatment delivery, audio recordings of three therapy sessions (early, middle and later sessions) for each participant will be rated by expert supervisors for therapist empathy using the Therapist Empathy Scale [29] and therapist fidelity to the protocol.

#### *Sample size*

To determine sample size, we carried out a ‘liberal’ power calculation for efficacy signal detection: In our pre-pilot uncontrolled feasibility study, we achieved clinically worthwhile reductions in the Time Budget of 0.83 effect size (Cohen’s *d*) between pre- and post-therapy, which is in line with published effect sizes for these interventions in meta-analyses in nonpsychotic client populations (range from 1.6 to 0.6: [9,11]). We therefore estimate a change in Time Budget total scores from 52.3 to 61.8 (pooled standard deviation, 11.2) between baseline and post-treatment in the GOALS group and no change over time in the TAU group, an effect size of 0.8. A liberal two-sided *t* test at significance level 0.1 was used to assess power. A sample size of 56 (28 patients per group) would have greater than 90% power to detect this change. Recruiting to *N* = 66 in total will allow for 15% missing data or other loss to follow-up. Minimizing such losses and achieving a modest increase in recruitment by a further 12 participants would also have 90% power to demonstrate a statistically significant effect at the conventional 5% significance level.

#### *Randomization*

Following informed consent and completion of baseline assessments, individuals will be randomized to one of the treatment arms by the study coordinator. This will be conducted independently of the trial team by the King’s Clinical Trials Unit, using an online randomization system. Randomization will be carried out at a 50:50 ratio and will be at the level of the individual and stratified by main problem focus (anxiety; depression), using random permuted blocks (varying in size from three to six).

#### *Blinding*

To minimize systematic bias, all study members (including the statistician and assistant psychologists conducting the assessments) will be blind to treatment allocation, with the exception of the study coordinator who will be responsible for the allocation of therapy and clinical supervision. For any breaks in blindness, another assistant psychologist will be allocated to complete the next set of outcome measures where possible. If this is not possible for any reason, this will be recorded in the trial master file. Treatment-group-specific information, such as measures describing the trained staff therapists or aspects of the therapy, will be stored in a separate database and will only be made available to those analyzing the data once the blinded analyses have been completed.

#### *Data monitoring*

Data will be entered into an SPSS spreadsheet by the study researchers. There will be three separate spreadsheets for each assessment time point (0, 12 and 18 weeks). All data will be double-checked monthly for accuracy, referring back to the paper-based forms. The data will be locked following the completion and checking of the last participant at the final time point. The three separate SPSS spreadsheets for each assessment time point will be merged, once all data have been entered and checked. The randomization data and trial data will only be linked once all trial data collection is complete and the database is locked. All databases will be organized according to unique participant ID number (assigned at the point of randomization), which will ensure that the databases are merged correctly.

#### *Assessment of safety*

Serious adverse events and reactions will be monitored and recorded throughout the study period, from consent to the final follow-up assessment meeting. Assistant psychologists will assess any serious adverse events occurring over the course of the study at 12 weeks and 18 weeks. In addition, the study coordinator will review participants’ clinical notes for additional information regarding serious adverse events. Should any additional information arise, this will be recorded. The local NHS trust procedures for grading serious untoward incidents will be used. All serious untoward incidents are recorded as part of trust policy on clients’ electronic notes. Serious untoward incidents graded A to C will be classed as adverse events or reactions, which are defined as any events that result in death, serious injury or hospitalization. No serious adverse events are expected to occur as a result of the intervention; however, this will be monitored carefully and any events will be reported to the local research and development office by the study coordinator.

### Analysis

#### *Statistical analysis*

The outcomes of most interest are successful recruitment, randomization, quality and fidelity of the delivery of treatment and participant’s compliance with the protocol, including both treatment adherence and completion of research assessments. Descriptive statistics will be used to summarize these feasibility aspects and confidence intervals will be constructed for respective performance indices.

However, we will also carry out some efficacy comparisons of CBT + TAU versus TAU. These analyses will adhere to the intention-to-treat principle, with data from all participants entered into the analysis, including from all those who drop out of therapy (that is, those who do not attend at all or who cease therapy) or who miss some therapy sessions. Every effort will be made to follow up all participants in both arms for research assessments, and the analysis will use, where appropriate, statistical techniques for handling missing data. Linear mixed modelling will be used to compare mean outcomes between the treatment arms at each of the two post-treatment time points. More specifically, the outcome measured at the post-treatment time points will feature as the dependent variable and stratifier (main problem focus), pre-randomization outcome measure score, time, treatment arm and time × arm interaction term as explanatory variables. Correlation between the two repeated measures per person will be taken into account by including a subject varying random intercept. Therapist effects will be modelled by a further random intercept term that varies at the level of the therapist. A detailed statistical analysis plan has been written by the trial statistician.

If noncompliance with the active treatment (GOALS) is high, a complier average causal effect analysis will be considered, to estimate efficacy [30]. This might be warranted, since an intention-to-treat analysis estimates the effect of randomization group; that is, the treatment’s effectiveness only equals the treatment’s efficacy if every participant also receives the treatment that is allocated to them. Conventional per-protocol or as-treated analyses have long tried to address this but are subject to selection bias.

#### *Health economic analysis*

Service use is to be measured with the Client Service Receipt Inventory. Costs will be calculated by combining service use data with available unit cost information. The following comparisons between the groups at 12- and 18-weeks will be assessed: (i) proportion of patients using each service included in the Client Service Receipt Inventory; (ii) mean number of contacts with each service for those with at least one contact; (iii) mean cost of each service (including those with no contacts); (iv) mean total cost.

The first three comparisons will be descriptive with no tests of significance. The main focus will be on the comparison of total costs and for this we will use a regression model with total cost as the dependent variable, the group identifier variable as an independent variable, and baseline costs as an additional independent variable. We will visually check the distribution of the regression residuals; if these do not approximate a normal distribution, we will use bootstrapping methods with 1,000 repetitions to generate 95% confidence intervals.

A cost-consequences analysis will be conducted by viewing costs alongside efficacy measures. Cost effectiveness will be assessed by combining costs with the primary outcome of activity levels, using cost effectiveness planes, which will indicate the probability that the intervention is (i) cost saving with better outcomes, (ii) cost saving with worse outcomes, (iii) cost increasing with worse outcomes or (iv) cost increasing with better outcomes. These will be constructed by saving the 1,000 bootstrapped regression coefficients representing cost differences described previously and 1,000 bootstrapped regression coefficients representing outcome differences (change in activity levels) and plotting these against each other as a scatterplot.

#### *Qualitative analysis*

All interviews will be audiotaped, transcribed and analyzed qualitatively using thematic analysis [31], and indexed using NVivo software. Interviews conducted by study assistant psychologists and service user consultants will be analyzed separately, but outcomes will be compared. Thematic analysis has a number of key steps, including generating initial data-driven codes, searching and refining key themes, and defining and naming these themes before summarizing and producing a final report. To check reliability, at least 15% of the transcriptions will be second-coded and checked for agreement, to ensure that the coding framework is deemed reliable (at least 80% agreement).

## Discussion

Anxiety and depression are commonly present in people with psychosis and lead to inactivity and difficulties in achieving personal goals. Cognitive behavioural therapy for psychosis, which can be effective for emotional problems in psychosis [5,6,32], is a formulation-based therapy, delivered by highly trained therapists over 16 to 20 sessions, and, although recommended by NICE, is not widely available [33]. There are, however, effective brief protocol-based interventions, graded exposure and behavioural activation, which use cognitive behavioural principles and methods, for these problems. We have adapted these to address the specific needs and problems of people with psychosis, while ensuring a focus on personal recovery goals, and achieved promising results in our pre-pilot study [15]. The advantages of the GOALS approach are that it is brief and that the frontline mental health workers who are in regular contact with people with psychosis in care settings can be readily trained in its delivery. There is therefore scope for developing an effective intervention, which can be made widely available at low cost, improving access to psychological therapies for this client group. This is the first trial of the GOALS intervention. The study also aims to finalise the training curriculum and the treatment manual, which we intend to publish and make available in electronic form in the future. The trial is funded until August 2015 and the results will be available in 2016. If this intervention proves clinically feasible and demonstrates preliminary evidence of efficacy, a large multi-site trial will be warranted.

## Trial status

Participants began to enter the trial in April 2013. Recruitment will continue until December 2014.

## Abbreviations

CBT: cognitive behaviour therapy; GOALS: ‘Getting On top of Anxiety and Low mood’; NHS: National Health Service; NICE: National Institute for Health and Care Excellence; SMART: specific, measurable attainable, relevant and time-bound; TAU: treatment as usual.

## Competing interests

The authors declare that they have no competing interests.

## Authors’ contributions

PG and TC are the joint principal investigators for the study. HW is the study coordinator and drafted the study protocol. SL is the trial statistician; PM will lead the health economic analysis. NH and CI are the study assistant psychologists and are responsible for the recruitment of participants and data collection. MF is the lead for the study in early-intervention services. SJ is the lead for patient and public involvement in the study. All authors contributed to the design of the study protocol and were involved in writing the manuscript.
